# Supplementary material for: The unreliability of estimated release dates in hospital drug shortage management: A case study of hospital pharmacy operations during the COVID-19 pandemic
Source: PLoS One. 2025 Oct 13;20(10):e0328747. doi: 10.1371/journal.pone.0328747 (PMC12517479; doi:10.1371/journal.pone.0328747)
Supplement: Attachment — Submitted filename: Response to Reviewers.pdf [file pone.0328747.s001.pdf]

Dear Academic Editor and Reviewers,

First, I'd like to thank you all for taking the time to review our manuscript. We appreciate the opportunity to revise and resubmit, and your feedback was very helpful in improving the quality of the paper prior to publication.

Below are our responses to all the points raised by the editor and reviewers:

Main points:

1. There are some minor grammatical errors (duplicated words, i.e. the the).

Thank you for pointing these out. The fixes for these instances, along with other grammatical fixes, are highlighted in red in the track changes version of the manuscript.

2. Line 48: I am not sure which figure you are referencing by ??. Same thing in line 139, 183, 187, 192, 193, 199, etc. Following the paper was made hard because the figures weren't labelled correctly.

Our apologies for overlooking this. When removing the Figures from the original manuscript, we accidentally removed captions and labels as well, resulting in the question marks. The new version has the correct references and captions in the text. The figure files have also been renamed to more easily map them to each caption in the text.

3. Line 56: "First, if an ERD is far away" – the grammar is incorrect. Please review the paper with someone proficient in scientific English to improve the grammar to make the paper suitable for publication.

This has been changed in the text to "First, if an ERD is far in the future…". An ERD itself is a date, so we attempt to treat it as such grammatically throughout the manuscript.

4. Have you thought of developing an AI model to more accurately predict ERDs?

We chose to examine ERD accuracy and ERD changes statistically as a precursor for the development of operations management models. ERDs are just one piece of the larger drug shortage management problem which involves information, the costs of mitigation strategies, and coordination and trust among staff. Though we agree that AI would be a useful tool for accurately predicting shipment times given ERDs, it would not fully address the larger problem of consistently mitigating drug shortages in a manner that preserves patient health care quality and decreases labor and financial burdens on hospitals. This statistical analysis provides useful parameters and statistical tendencies for developing operations management models, which a predictive AI model would not necessarily provide.

5. Line 144: Please define all acronyms, such as NDC, ASHP, FDC, WSR, etc. Some readers may not be familiar with them, and they may not understand your paper.

Thank you for pointing this out. All acronyms are now defined in the first instances where they are encountered in the manuscript.

6. Table 2: Drug class: numbering goes from 1, 2, to 4, skipping 3. Were there really this few drug classes?

This error in the table has been corrected to number the drug class groups 1, 2, and 3. There were 37 different drug classes represented in the dataset. However, to ensure robustness of statistical tests, we grouped together all the classes that had 10 or fewer data points. Only 2 of the 37 drug classes had at least 11 data points, which are the two distinct groups (amide local anesthetics and nucleoside metabolic inhibitors) that are not included in the “Other” grouping. The “Other” group contains all the data points representing the other 35 drug classes. We added details to Section 3 to make the reasoning for this choice clearer.

7. Lines 207-209: “Additionally, a KW test concluded that these distributions do not significantly differ from one another (p = 0.223), suggesting that ERD accuracy does not statistically improve as ERDs are updated.” In Figure c1-r-fig1b, the 11-15 week ERD magnitude group seems to be significantly different than the other three groups. How do you account for this?

Figure c1-r-fig1b (now Figure 3b), shows plots for shipment lateness based on the magnitude of the original ERD (without considering any ERD changes). A KW test for this plot showed that the 11-15 ERD magnitude group was different from the others, suggesting that shipments arrive sooner than the ERDs in this group.

The KW test mentioned in lines 207-209 is referring to the distributions seen in Figure 5c. This figure shows the shipment lateness for all data points where the ERD changed 1, 2, 3, or 4 times. The KW test showed that the shipment lateness did not improve as the ERD was changed 1, 2, 3, or 4 times before the shipment arrived.

These two plots and statistical tests test two independent phenomenon (original ERD lateness vs. ERD lateness when the ERD changes). We hope that the inclusion of figure captions and properly labeled figures helps clear up the independence of these two results.

8. Lines 249-252: did you do a drill down of the data to see whether different contracts, geography or ordering behavior differ in ERD? This might uncover some factor that causes inaccurate ERDs.

The data we collected from our hospital collaborator did not include contractual, geographical, or ordering data, which is why we kept the scope to only manufacturer, drug class, and drug packaging groups.

Section 5.1 (Limitations) has been expanded to discuss the need for this exploration in future research.

<u>Reviewer Comments:</u>

Reviewer 1:

However, the study is largely descriptive, based on one well-resourced hospital, which limits generalizability. Costs are discussed qualitatively, without economic analysis, and the real impact on patient outcomes is not measured. ERD reliability is identified as an issue but not

explored with data. The perspective is narrow, focusing mainly on pharmacy managers while excluding voices from clinicians, procurement staff, or patients.

From a low- and middle-income country (LMIC) viewpoint, direct implementation of these strategies is challenging. Many LMIC hospitals lack in-house compounding facilities, real-time inventory systems, or direct manufacturer links. Supply chains are often dominated by distributors and use of alternative stock.

During COVID-19, LMIC hospitals faced similar surges in demand but with added constraints: import delays, market hoarding, and no central shortage reporting system. Shortage responses were often informal — relying on phone calls, personal networks, and improvised substitutions — rather than structured protocols.

Overall, the article is a valuable case study for high-resource settings but needs adaptation to be relevant in LMIC contexts.

Broader stakeholder perspectives, data on ERD accuracy, and examples from smaller or less resourced hospitals would make it more globally applicable.

There are a variety of stakeholders that are involved in and affected by drug shortage management decisions beyond pharmacy managers, such as procurement staff, physicians, patients, and more. We attempt to simplify the language in the manuscript by using the phrase “pharmacy managers” throughout. We have added this acknowledgement in Section 1.1.

Additionally, these are all crucial limitations of this particular case study. We have expanded Section 5.1 (Limitations) to elaborate on the topics of financial resources and emphasize that this is a case study of one, well-resourced hospital, limiting its broader applicability to others.

Reviewer 2:

The only observations that should be corrected: line 18 – “from” repeats consecutively, line 48, 139, 183, 187, 192, 193, 199 – refers to Figure ??.

We apologize for this oversight. We have fixed the manuscript so that figure references appear correctly and figure captions are included. Figure file names were also changed to directly correspond to the figure number in the text.

Reviewer 3:

However, the dataset is limited to a single hospital (understood for the confidentiality reasons), and more context on hospital size or setting would help readers assess generalizability. I also encourage the authors to expand discussion of potential factor that may drive ERD inaccuracy.

Our hospital collaborators chose to remain anonymous for this study. However, we changed Section 1 to include details that the hospital has >500 staffed beds, providing additional context for the reader.

We have revised Section 5.1 (Limitations) to include additional factors that could contribute to ERD inaccuracy, like geography and supply chain structure. Also, we mention specific supply chain factors, including variable shipment lead times upon release, wholesalers not fulfilling

every hospital's back order upon each release, or the difficulty involved in manufacturers accurately predicting the date of future releases in Section 5. We chose to not elaborate more on these potential factors or other factors to avoid speculation without evidence.

We have attached a track changes version of the manuscript that contains all of the changes discussed above, along with a clean version incorporating these changes. The changes in the tracked version are colored in red.

Please let me know if any additional formatting or content changes are required. And thank you all again for reviewing and providing feedback on this manuscript.

Sincerely,
Noah Chicoine
chicoine.n@northeastern.edu
