# Supplementary material for: The unreliability of estimated release dates in hospital drug shortage management: A case study of hospital pharmacy operations during the COVID-19 pandemic
Source: PLoS One. 2025 Oct 13;20(10):e0328747. doi: 10.1371/journal.pone.0328747 (PMC12517479; doi:10.1371/journal.pone.0328747)
Supplement: Peer review history

# Peer review: Decision Letter 0

**DOI:** 10.1371/journal.pone.0328747.r001

**Authors:** Matthew Cserhati

20 Aug 2025

PONE-D-25-35006The Unreliability of Estimated Release Dates in Hospital Drug Shortage Management: A Case Study of Hospital Pharmacy Operations During the COVID-19 PandemicPLOS ONE

Dear Dr. Chicoine,

Thank you for submitting your manuscript to PLOS ONE. After careful consideration, we feel that it has merit but does not fully meet PLOS ONE’s publication criteria as it currently stands. Therefore, we invite you to submit a revised version of the manuscript that addresses the points raised during the review process.

==============================

1. There are some minor grammatical errors (duplicated words, i.e. the the).
2. Line 48: I am not sure which figure you are referencing by ??. Same thing in line 139, 183, 187, 192, 193, 199, etc. Following the paper was made hard because the figures weren’t labelled correctly.
3. Line 56: **“First, if an ERD is far away”** – the grammar is incorrect. Please review the paper with someone proficient in scientific English to improve the grammar to make the paper suitable for publication.
4. Have you thought of developing an AI model to more accurately predict ERDs?
5. Line 144: Please define all acronyms, such as NDC, ASHP, FDC, WSR, etc. Some readers may not be familiar with them, and they may not understand your paper.
6. Table 2: Drug class: numbering goes from 1, 2, to 4, skipping 3. Were there really this few drug classes?
7. **Lines 207-209: “Additionally, a KW test concluded that  these distributions do not significantly differ from one another (p = 0.223), suggesting that ERD accuracy does not statistically improve as ERDs are updated.”**

In Figure c1-r-fig1b, the 11-15 week ERD magnitude group seems to be significantly different than the other three groups. How do you account for this?

1. Lines 249-252: did you do a drill down of the data to see whether different contracts, geography or ordering behavior differ in ERD? This might uncover some factor that causes inaccurate ERDs.

==============================

Please submit your revised manuscript by Oct 04 2025 11:59PM. If you will need more time than this to complete your revisions, please reply to this message or contact the journal office at plosone@plos.org. When you're ready to submit your revision, log on to https://www.editorialmanager.com/pone/ and select the 'Submissions Needing Revision' folder to locate your manuscript file.

Please include the following items when submitting your revised manuscript:

- A rebuttal letter that responds to each point raised by the academic editor and reviewer(s). You should upload this letter as a separate file labeled 'Response to Reviewers'.
- A marked-up copy of your manuscript that highlights changes made to the original version. You should upload this as a separate file labeled 'Revised Manuscript with Track Changes'.
- An unmarked version of your revised paper without tracked changes. You should upload this as a separate file labeled 'Manuscript'.

If you would like to make changes to your financial disclosure, please include your updated statement in your cover letter. Guidelines for resubmitting your figure files are available below the reviewer comments at the end of this letter.

If applicable, we recommend that you deposit your laboratory protocols in protocols.io to enhance the reproducibility of your results. Protocols.io assigns your protocol its own identifier (DOI) so that it can be cited independently in the future. For instructions see: https://journals.plos.org/plosone/s/submission-guidelines#loc-laboratory-protocols. Additionally, PLOS ONE offers an option for publishing peer-reviewed Lab Protocol articles, which describe protocols hosted on protocols.io. Read more information on sharing protocols at https://plos.org/protocols?utm_medium=editorial-email&utm_source=authorletters&utm_campaign=protocols.

We look forward to receiving your revised manuscript.

Kind regards,

Matthew Cserhati, Ph.D

Academic Editor

PLOS ONE

Journal Requirements: 

When submitting your revision, we need you to address these additional requirements.

1. Please ensure that your manuscript meets PLOS ONE's style requirements, including those for file naming. The PLOS ONE style templates can be found at

https://journals.plos.org/plosone/s/file?id=wjVg/PLOSOne_formatting_sample_main_body.pdf and

https://journals.plos.org/plosone/s/file?id=ba62/PLOSOne_formatting_sample_title_authors_affiliations.pdf.

2. Please update your submission to use the PLOS LaTeX template. The template and more information on our requirements for LaTeX submissions can be found at http://journals.plos.org/plosone/s/latex.

3. We note that the grant information you provided in the ‘Funding Information’ and ‘Financial Disclosure’ sections do not match.

When you resubmit, please ensure that you provide the correct grant numbers for the awards you received for your study in the ‘Funding Information’ section.

4. Thank you for stating the following financial disclosure:

 [This work was done in partial support of NSF RAPID Grant #2028449 and NSF SAI Grant #2228510]. 

Please state what role the funders took in the study.  If the funders had no role, please state: ""The funders had no role in study design, data collection and analysis, decision to publish, or preparation of the manuscript.""

If this statement is not correct you must amend it as needed.

Please include this amended Role of Funder statement in your cover letter; we will change the online submission form on your behalf.

5. Please expand the acronym “NSF” (as indicated in your financial disclosure) so that it states the name of your funders in full.

This information should be included in your cover letter; we will change the online submission form on your behalf.

6. Thank you for stating the following in the Acknowledgments Section of your manuscript:

[This work was funded partially by NSF RAPID Grant #2028449 and NSF SAI Grant #2228510.]

We note that you have provided funding information that is not currently declared in your Funding Statement. However, funding information should not appear in the Acknowledgments section or other areas of your manuscript. We will only publish funding information present in the Funding Statement section of the online submission form.

Please remove any funding-related text from the manuscript and let us know how you would like to update your Funding Statement. Currently, your Funding Statement reads as follows:

  [This work was done in partial support of NSF RAPID Grant #2028449 and NSF SAI Grant #2228510]. 

Please include your amended statements within your cover letter; we will change the online submission form on your behalf.

7.  Please amend either the abstract on the online submission form (via Edit Submission) or the abstract in the manuscript so that they are identical.

8.  Please include a caption for figure 1 to 4.

9. We note that you have indicated that there are restrictions to data sharing for this study. For studies involving human research participant data or other sensitive data, we encourage authors to share de-identified or anonymized data. However, when data cannot be publicly shared for ethical reasons, we allow authors to make their data sets available upon request. For information on unacceptable data access restrictions, please see http://journals.plos.org/plosone/s/data-availability#loc-unacceptable-data-access-restrictions.

Before we proceed with your manuscript, please address the following prompts:

a) If there are ethical or legal restrictions on sharing a de-identified data set, please explain them in detail (e.g., data contain potentially identifying or sensitive patient information, data are owned by a third-party organization, etc.) and who has imposed them (e.g., a Research Ethics Committee or Institutional Review Board, etc.). Please also provide contact information for a data access committee, ethics committee, or other institutional body to which data requests may be sent.

b) If there are no restrictions, please upload the minimal anonymized data set necessary to replicate your study findings to a stable, public repository and provide us with the relevant URLs, DOIs, or accession numbers. Please see http://www.bmj.com/content/340/bmj.c181.long for guidelines on how to de-identify and prepare clinical data for publication. For a list of recommended repositories, please see https://journals.plos.org/plosone/s/recommended-repositories. You also have the option of uploading the data as Supporting Information files, but we would recommend depositing data directly to a data repository if possible.

Please update your Data Availability statement in the submission form accordingly.

10. If the reviewer comments include a recommendation to cite specific previously published works, please review and evaluate these publications to determine whether they are relevant and should be cited. There is no requirement to cite these works unless the editor has indicated otherwise. 

11. Please review your reference list to ensure that it is complete and correct. If you have cited papers that have been retracted, please include the rationale for doing so in the manuscript text, or remove these references and replace them with relevant current references. Any changes to the reference list should be mentioned in the rebuttal letter that accompanies your revised manuscript. If you need to cite a retracted article, indicate the article’s retracted status in the References list and also include a citation and full reference for the retraction notice.

Additional Editor Comments:

1. There are some minor grammatical errors (duplicated words, i.e. the the).

2. Line 48: I am not sure which figure you are referencing by ??. Same thing in line 139, 183, 187, 192, 193, 199, etc. Following the paper was made hard because the figures weren’t labelled correctly.

3. Line 56: “First, if an ERD is far away” – the grammar is incorrect. Please review the paper with someone proficient in scientific English to improve the grammar to make the paper suitable for publication.

4. Have you thought of developing an AI model to more accurately predict ERDs?

5. Line 144: Please define all acronyms, such as NDC, ASHP, FDC, WSR, etc. Some readers may not be familiar with them, and they may not understand your paper.

6. Table 2: Drug class: numbering goes from 1, 2, to 4, skipping 3. Were there really this few drug classes?

7. Lines 207-209: “Additionally, a KW test concluded that these distributions do not significantly differ from one another (p = 0.223), suggesting that ERD accuracy does not statistically improve as ERDs are updated.”

In Figure c1-r-fig1b, the 11-15 week ERD magnitude group seems to be significantly different than the other three groups. How do you account for this?

8. Lines 249-252: did you do a drill down of the data to see whether different contracts, geography or ordering behavior differ in ERD? This might uncover some factor that causes inaccurate ERDs.

[Note: HTML markup is below. Please do not edit.]

Reviewers' comments:

Reviewer's Responses to Questions

**Comments to the Author**

1. Is the manuscript technically sound, and do the data support the conclusions?

The manuscript must describe a technically sound piece of scientific research with data that supports the conclusions. Experiments must have been conducted rigorously, with appropriate controls, replication, and sample sizes. The conclusions must be drawn appropriately based on the data presented. 

Reviewer #1: Yes

Reviewer #2: Yes

Reviewer #3: Yes

**********

2. Has the statistical analysis been performed appropriately and rigorously? 

Reviewer #1: Yes

Reviewer #2: Yes

Reviewer #3: Yes

**********

3. Have the authors made all data underlying the findings in their manuscript fully available?

The PLOS Data policy requires authors to make all data underlying the findings described in their manuscript fully available without restriction, with rare exception (please refer to the Data Availability Statement in the manuscript PDF file). The data should be provided as part of the manuscript or its supporting information, or deposited to a public repository. For example, in addition to summary statistics, the data points behind means, medians and variance measures should be available. If there are restrictions on publicly sharing data—e.g. participant privacy or use of data from a third party—those must be specified.

Reviewer #1: Yes

Reviewer #2: Yes

Reviewer #3: No

**********

4. Is the manuscript presented in an intelligible fashion and written in standard English?

PLOS ONE does not copyedit accepted manuscripts, so the language in submitted articles must be clear, correct, and unambiguous. Any typographical or grammatical errors should be corrected at revision, so please note any specific errors here.

Reviewer #1: Yes

Reviewer #2: Yes

Reviewer #3: Yes

**********

5. Review Comments to the Author

Please use the space provided to explain your answers to the questions above. You may also include additional comments for the author, including concerns about dual publication, research ethics, or publication ethics. (Please upload your review as an attachment if it exceeds 20,000 characters)

Reviewer #1: This article provides a practical picture of how a large U.S. hospital managed drug shortages during COVID-19. Its main strength is the detailed description of the workflow — from stock checks and supply chain details to substitution decisions — and the clear table of mitigation strategies. Highlighting the role of “estimated release dates” (ERDs) from manufacturers adds value, as it touches on a critical but often overlooked part of shortage management.

However, the study is largely descriptive, based on one well-resourced hospital, which limits generalizability. Costs are discussed qualitatively, without economic analysis, and the real impact on patient outcomes is not measured. ERD reliability is identified as an issue but not explored with data. The perspective is narrow, focusing mainly on pharmacy managers while excluding voices from clinicians, procurement staff, or patients.

From a low- and middle-income country (LMIC) viewpoint, direct implementation of these strategies is challenging. Many LMIC hospitals lack in-house compounding facilities, real-time inventory systems, or direct manufacturer links. Supply chains are often dominated by distributors and use of alternative stock.

During COVID-19, LMIC hospitals faced similar surges in demand but with added constraints: import delays, market hoarding, and no central shortage reporting system. Shortage responses were often informal — relying on phone calls, personal networks, and improvised substitutions — rather than structured protocols.

Overall, the article is a valuable case study for high-resource settings but needs adaptation to be relevant in LMIC contexts.

Broader stakeholder perspectives, data on ERD accuracy, and examples from smaller or less resourced hospitals would make it more globally applicable.

Reviewer #2: The paper is well-organized with each section properly separated. The authors provide a good summary of literature regarding the research topic. The aim of the study was clearly presented. The methodology is clear and can be replicated. The statistical analysis is complete and presented in a clear format with adequate explanations to understand the results. The results are presented clearly and in an organized manner. The authors discussed in detail the results and the implications of their study. Overall, is well-thought-out with successful execution presented in a clear manner. The only observations that should be corrected: line 18 – “from” repeats consecutively, line 48, 139, 183, 187, 192, 193, 199 – refers to Figure ??.

Reviewer #3: This is a novel and significant study, providing the first empirical evidence that Estimated Release Dates (ERDs) are unreliable for hospital drug shortage management. The analysis is rigorous and the findings have clear practical importance.

However, the dataset is limited to a single hospital (understood for the confidentiality reasons), and more context on hospital size or setting would help readers assess generalizability. I also encourage the authors to expand discussion of potential factor that may drive ERD inaccuracy.

**********

6. PLOS authors have the option to publish the peer review history of their article (what does this mean?). If published, this will include your full peer review and any attached files.

If you choose “no”, your identity will remain anonymous but your review may still be made public.

**Do you want your identity to be public for this peer review?** For information about this choice, including consent withdrawal, please see our Privacy Policy.

Reviewer #1: **Yes: **Sadia Sultana Resma

Reviewer #2: **Yes: **Isabel R Pogozelski, DNP, FNP-BC

Reviewer #3: No

**********

[NOTE: If reviewer comments were submitted as an attachment file, they will be attached to this email and accessible via the submission site. Please log into your account, locate the manuscript record, and check for the action link "View Attachments". If this link does not appear, there are no attachment files.]

While revising your submission, please upload your figure files to the Preflight Analysis and Conversion Engine (PACE) digital diagnostic tool, https://pacev2.apexcovantage.com/. PACE helps ensure that figures meet PLOS requirements. To use PACE, you must first register as a user. Registration is free. Then, login and navigate to the UPLOAD tab, where you will find detailed instructions on how to use the tool. If you encounter any issues or have any questions when using PACE, please email PLOS at figures@plos.org. Please note that Supporting Information files do not need this step.

# Author comment: Author response to Decision Letter 1

**DOI:** 10.1371/journal.pone.0328747.r002

12 Sep 2025

Main points:

1. There are some minor grammatical errors (duplicated words, i.e. the the).

Thank you for pointing these out. The fixes for these instances, along with other grammatical fixes, are highlighted in red in the track changes version of the manuscript.

2. Line 48: I am not sure which figure you are referencing by ??. Same thing in line 139, 183, 187, 192, 193, 199, etc. Following the paper was made hard because the figures weren’t labelled correctly.

Our apologies for overlooking this. When removing the Figures from the original manuscript, we accidentally removed captions and labels as well, resulting in the question marks. The new version has the correct references and captions in the text. The figure files have also been renamed to more easily map them to each caption in the text.

3. Line 56: “First, if an ERD is far away” – the grammar is incorrect. Please review the paper with someone proficient in scientific English to improve the grammar to make the paper suitable for publication.

This has been changed in the text to “First, if an ERD is far in the future…”. An ERD itself is a date, so we attempt to treat it as such grammatically throughout the manuscript.

4. Have you thought of developing an AI model to more accurately predict ERDs?

We chose to examine ERD accuracy and ERD changes statistically as a precursor for the development of operations management models. ERDs are just one piece of the larger drug shortage management problem which involves information, the costs of mitigation strategies, and coordination and trust among staff. Though we agree that AI would be a useful tool for accurately predicting shipment times given ERDs, it would not fully address the larger problem of consistently mitigating drug shortages in a manner that preserves patient health care quality and decreases labor and financial burdens on hospitals. This statistical analysis provides useful parameters and statistical tendencies for developing operations management models, which a predictive AI model would not necessarily provide.

5. Line 144: Please define all acronyms, such as NDC, ASHP, FDC, WSR, etc. Some readers may not be familiar with them, and they may not understand your paper.

Thank you for pointing this out. All acronyms are now defined in the first instances where they are encountered in the manuscript.

6. Table 2: Drug class: numbering goes from 1, 2, to 4, skipping 3. Were there really this few drug classes?

This error in the table has been corrected to number the drug class groups 1, 2, and 3. There were 37 different drug classes represented in the dataset. However, to ensure robustness of statistical tests, we grouped together all the classes that had 10 or fewer data points. Only 2 of the 37 drug classes had at least 11 data points, which are the two distinct groups (amide local anesthetics and nucleoside metabolic inhibitors) that are not included in the “Other” grouping. The “Other” group contains all the data points representing the other 35 drug classes. We added details to Section 3 to make the reasoning for this choice clearer.

7. Lines 207-209: “Additionally, a KW test concluded that these distributions do not significantly differ from one another (p = 0.223), suggesting that ERD accuracy does not statistically improve as ERDs are updated.” In Figure c1-r-fig1b, the 11-15 week ERD magnitude group seems to be significantly different than the other three groups. How do you account for this?

Figure c1-r-fig1b (now Figure 3b), shows plots for shipment lateness based on the magnitude of the original ERD (without considering any ERD changes). A KW test for this plot showed that the 11-15 ERD magnitude group was different from the others, suggesting that shipments arrive sooner than the ERDs in this group.

The KW test mentioned in lines 207-209 is referring to the distributions seen in Figure 5c. This figure shows the shipment lateness for all data points where the ERD changed 1, 2, 3, or 4 times. The KW test showed that the shipment lateness did not improve as the ERD was changed 1, 2, 3, or 4 times before the shipment arrived.

These two plots and statistical tests test two independent phenomenon (original ERD lateness vs. ERD lateness when the ERD changes). We hope that the inclusion of figure captions and properly labeled figures helps clear up the independence of these two results.

8. Lines 249-252: did you do a drill down of the data to see whether different contracts, geography or ordering behavior differ in ERD? This might uncover some factor that causes inaccurate ERDs.

The data we collected from our hospital collaborator did not include contractual, geographical, or ordering data, which is why we kept the scope to only manufacturer, drug class, and drug packaging groups.

Section 5.1 (Limitations) has been expanded to discuss the need for this exploration in future research.

Reviewer Comments:

Reviewer 1:

However, the study is largely descriptive, based on one well-resourced hospital, which limits generalizability. Costs are discussed qualitatively, without economic analysis, and the real impact on patient outcomes is not measured. ERD reliability is identified as an issue but not explored with data. The perspective is narrow, focusing mainly on pharmacy managers while excluding voices from clinicians, procurement staff, or patients.

From a low- and middle-income country (LMIC) viewpoint, direct implementation of these strategies is challenging. Many LMIC hospitals lack in-house compounding facilities, real-time inventory systems, or direct manufacturer links. Supply chains are often dominated by distributors and use of alternative stock.

During COVID-19, LMIC hospitals faced similar surges in demand but with added constraints: import delays, market hoarding, and no central shortage reporting system. Shortage responses were often informal — relying on phone calls, personal networks, and improvised substitutions — rather than structured protocols.

Overall, the article is a valuable case study for high-resource settings but needs adaptation to be relevant in LMIC contexts.

Broader stakeholder perspectives, data on ERD accuracy, and examples from smaller or less resourced hospitals would make it more globally applicable.

--

There are a variety of stakeholders that are involved in and affected by drug shortage management decisions beyond pharmacy managers, such as procurement staff, physicians, patients, and more. We attempt to simplify the language in the manuscript by using the phrase “pharmacy managers” throughout. We have added this acknowledgement in Section 1.1.

Additionally, these are all crucial limitations of this particular case study. We have expanded Section 5.1 (Limitations) to elaborate on the topics of financial resources and emphasize that this is a case study of one, well-resourced hospital, limiting its broader applicability to others.

Reviewer 2:

The only observations that should be corrected: line 18 – “from” repeats consecutively, line 48, 139, 183, 187, 192, 193, 199 – refers to Figure ??.

--

We apologize for this oversight. We have fixed the manuscript so that figure references appear correctly and figure captions are included. Figure file names were also changed to directly correspond to the figure number in the text.

Reviewer 3:

However, the dataset is limited to a single hospital (understood for the confidentiality reasons), and more context on hospital size or setting would help readers assess generalizability. I also encourage the authors to expand discussion of potential factor that may drive ERD inaccuracy.

--

Our hospital collaborators chose to remain anonymous for this study. However, we changed Section 1 to include details that the hospital has >500 staffed beds, providing additional context for the reader.

We have revised Section 5.1 (Limitations) to include additional factors that could contribute to ERD inaccuracy, like geography and supply chain structure. Also, we mention specific supply chain factors, including variable shipment lead times upon release, wholesalers not fulfilling every hospital's back order upon each release, or the difficulty involved in manufacturers accurately predicting the date of future releases in Section 5. We chose to not elaborate more on these potential factors or other factors to avoid speculation without evidence.

Attachment Submitted filename: Response to Reviewers.pdf

# Editor report: Decision Letter 1

**DOI:** 10.1371/journal.pone.0328747.r003

**Authors:** Matthew Cserhati

16 Sep 2025

The Unreliability of Estimated Release Dates in Hospital Drug Shortage Management: A Case Study of Hospital Pharmacy Operations During the COVID-19 Pandemic

PONE-D-25-35006R1

Dear Dr. Chicoine,

We’re pleased to inform you that your manuscript has been judged scientifically suitable for publication and will be formally accepted for publication once it meets all outstanding technical requirements.

Within one week, you’ll receive an e-mail detailing the required amendments. When these have been addressed, you’ll receive a formal acceptance letter and your manuscript will be scheduled for publication.

An invoice will be generated when your article is formally accepted. Please note, if your institution has a publishing partnership with PLOS and your article meets the relevant criteria, all or part of your publication costs will be covered. Please make sure your user information is up-to-date by logging into Editorial Manager at Editorial Manager® and clicking the ‘Update My Information' link at the top of the page. For questions related to billing, please contact billing support.

If your institution or institutions have a press office, please notify them about your upcoming paper to help maximize its impact. If they’ll be preparing press materials, please inform our press team as soon as possible -- no later than 48 hours after receiving the formal acceptance. Your manuscript will remain under strict press embargo until 2 pm Eastern Time on the date of publication. For more information, please contact onepress@plos.org.

Kind regards,

Matthew Cserhati, Ph.D

Academic Editor

PLOS ONE

Additional Editor Comments (optional):

1. Line 208: [NDC] should be (NDC).

Reviewers' comments:

# Editor report: Acceptance letter

**DOI:** 10.1371/journal.pone.0328747.r004

**Authors:** Matthew Cserhati

PONE-D-25-35006R1

PLOS ONE

Dear Dr. Chicoine,

I'm pleased to inform you that your manuscript has been deemed suitable for publication in PLOS ONE. Congratulations! Your manuscript is now being handed over to our production team.

At this stage, our production department will prepare your paper for publication. This includes ensuring the following:

* All references, tables, and figures are properly cited

* All relevant supporting information is included in the manuscript submission,

* There are no issues that prevent the paper from being properly typeset

You will receive further instructions from the production team, including instructions on how to review your proof when it is ready. Please keep in mind that we are working through a large volume of accepted articles, so please give us a few days to review your paper and let you know the next and final steps.

Lastly, if your institution or institutions have a press office, please let them know about your upcoming paper now to help maximize its impact. If they'll be preparing press materials, please inform our press team within the next 48 hours. Your manuscript will remain under strict press embargo until 2 pm Eastern Time on the date of publication. For more information, please contact onepress@plos.org.

You will receive an invoice from PLOS for your publication fee after your manuscript has reached the completed accept phase. If you receive an email requesting payment before acceptance or for any other service, this may be a phishing scheme. Learn how to identify phishing emails and protect your accounts at https://explore.plos.org/phishing.

If we can help with anything else, please email us at customercare@plos.org.

Thank you for submitting your work to PLOS ONE and supporting open access.

Kind regards,

PLOS ONE Editorial Office Staff

on behalf of

Dr. Matthew Cserhati

Academic Editor

PLOS ONE
